# Identification and characterization of the expression profile of microRNAs in *Anopheles anthropophagus*

**DOI:** 10.1186/1756-3305-7-159

**Published:** 2014-04-01

**Authors:** Wenquan Liu, Huicong Huang, Cuicui Xing, Chunxiang Li, Feng Tan, Shaohui Liang

**Affiliations:** 1 Department of Parasitology, Wenzhou Medical University, Wenzhou, Zhejiang Province 325035, PR China

**Keywords:** MicroRNAs (miRNAs), *Anopheles anthropophagus*, Expression profile, Sexual differences, Stage-specific

## Abstract

**Background:**

*Anopheles anthropophagus*, one of the most important mosquito-borne disease vectors in Asia, mainly takes blood meals from humans and transmits both malaria and filariae*.* MicroRNAs (miRNAs) are small non-coding RNAs, and play a critical role in many cellular processes, including development, differentiation, apoptosis and innate immunity.

**Methods:**

We investigated the global miRNA expression profile of male and female adults of *A. anthropophagus* using illumina Hiseq2000 sequencing combined with Northern blot.

**Results:**

By using the miRNAs of the closely-related species *Anopheles gambiae* and *Aedes aegypti* as reference, we obtained 102 miRNAs candidates out of 12.43 million raw sequencing reads for male and 16.51 million reads for female, with 81 of them found as known miRNAs in *An. gambiae* and/or *Ae. aegypti*, and the remaining 21 miRNAs were considered as novel. By analyzing the revised read count of miRNAs in male and female, 29 known miRNAs show sexual difference expression: >2-fold in the read count of the same miRNAs in male and female. Especially for miR-989, which is highly expressed in the female mosquitoes, but shows almost no detected expression in male mosquitoes, indicating that miR-989 may be involved in the physiological activity of female mosquito adults. The expression of four miRNAs in different growth stages of mosquito were further identified by Northern blot. Several miRNAs show the stage-specific expression, of which miR-2943 only expressed in the egg stage, suggesting that miR-2943 may be associated with the development of mosquito eggs.

**Conclusions:**

The present study represents the first global characterization of *An. anthropophagus* miRNAs in sexual differences and stage-specific functions. A better understanding of the functions of these miRNAs will offer new insights in mosquito biology and has implications for the effective control of mosquito-borne infectious diseases.

## Background

Mosquitoes are important vectors in the transmission of viruses and parasitic diseases, which represent an important global public health problem, especially in tropical and subtropical areas [1,2]. Due to the lack of an efficient vaccine and drug against the pathogens including plasmodium and dengue virus, mosquito control has been the primary strategy for preventing mosquito-borne disease [1,3]. *Anopheles anthropophagus* (*An. anthropophagus*), which belongs to the Hyrcanus group of the genus Anopheles, was identified as a major vector for malaria and filariasis in Asia [4].

MicroRNAs (miRNAs) are small (approximately 18–24 nucleotides in length), non-coding RNAs that are produced by all animals, plants and some viruses [5-7]. They regulate the expression of cellular genes by guiding the RNA-induced silencing complex (RISC) to target the 3′ UTR of mRNAs for cleavage or translational repression [8,9], and play a critical role in many cellular processes, including development, differentiation, apoptosis and innate immunity [10-14]. Undoubtedly, they are also implicated in the physiological functions of mosquitoes, such as sexual difference and blood feeding, even in the control of viral and parasitic infection [12-15].

Until now, thousands of miRNAs have been reported in animals, plants and viruses (miRBase: http://www.mirbase.org) [16]. Many miRNAs are highly conserved across divergent species while others are specific to a particular evolutionary lineage [13,15]. The miRNAs of some members of the mosquitoes, including *Anopheles gambiae*, *Aedes aegypti*, *Aedes albopictus*, *Culex quinquefasciatus* and *Anopheles stephensi* have been reported [13,15-21]. However, there was no miRNAs identified from *An. anthropophagus* despite its important role in transmitting parasitic disease.

In this study, we performed the first systematic analysis of miRNAs in *An. Anthropophagus* by using high throughput sequencing and bioinformatics approaches. Due to differences in feeding behaviours between the female and male adult mosquitoes, and female adults playing an important role in transmitting pathogens [22]. The miRNA expression in the sexual difference and specific- stage was substantially analyzed to study the potential role of miRNAs in development and physiological activity. Meanwhile, a number of novel *An. anthropophagus* mosquito-specific miRNAs were discovered. Our analysis also offered insights into the evolution of conserved and lineage-specific miRNAs in mosquitoes, and has implications for the effective control of mosquito-borne infectious diseases.

## Methods

### Source material and ethics statement

*Anopheles anthropophagus* (China wild type strain originally from Jiangsu Institute of parasitic diseases prevention, Jiangsu, P.R. China) were reared in a humidified insectary at 26 ± 1°C on a 12 hour light: dark cycle. Adult mosquitoes were kept in a 30 × 30 × 40 cm screened cages and provided constant access to water and glucose-soaked sponges.

ICR mice (Animal Experiment Centre of Wenzhou Medical University) were used in this study to offer a blood meal. The procedure was handled in accordance with good animal practices required by the Animal Ethics Procedures and Guidelines of the People’s Republic of China

### Total RNA isolation and small RNA library preparation

Total RNA was prepared from 100 adult male or female *An. anthropophagus* mosquitoes using trizol (Invitrogen) according to the manufacturer’s protocol. The isolation methods of total RNA and small RNA were unbiased in each sample. All samples were ground in liquid nitrogen and the quality of RNA was detected by using denaturalization agar gels and Du-530 Spectrophotometer (Beckman, Gemany).

The RNA smaller than 200 bp were enriched with the mirVana miRNA isolation kit (Ambion, USA). The small RNA samples were sent to Genergy Bio. (Shanghai, China) for small RNA cloning. The population of miRNAs with a length of 15–30 bp was passively eluted from polyacrylamide gels. The RNA was then precipitated with ethanol and dissolved in water. The small RNAs collected had a poly(A)- tail added to their 3'–OH by poly-(A) polymerase. The 5′-phosphate of the small RNAs were ligated to an RNA adapter. First-strand cDNA synthesis was then performed using an oligo(dT)-linker primer and MMLV-RNase H reverse transcriptase (Promega, USA). The resulting cDNAs were PCR amplified to ~ 25 μg/μl.

### High-throughput sequencing and computational analysis *An. anthropophagus* miRNAs

Primers used for PCR amplification were designed for amplicon sequencing according to the instructions of illumina Hiseq2000 (BGI, China). The PCR-amplified cDNAs were size-selected using electroelution to obtain products of 119–134 bp. These cDNAs were then sequenced by illumina Hiseq2000. Adaptors, low quality reads and reads smaller than 18 nucleotides (nt) were firstly removed from the total small RNA read datasets of male and female adults, respectively. No publically available genome is currently accessible for *An. anthropophagus*, the genome of the related mosquito and the closely related species in genetic distance *An. gambiae* and *Ae. aegypti* were used as a reference genome. The clean read datasets were blasted with BOWTIE software according to the following criteria: a 5′ and 3′ linker match of at least 15 nt and an appropriate length (18–28 nt). The pre-miRNAs and mature miRNAs in the miRBase v.20.0 were searched with BLAST software to identify *An. anthropophagus* miRNAs. Rfam (10.1) database (http://rfam.sanger.ac.uk/)was used to remove non-miRNAs, including rRNA, tRNA, snRNA, snoRNA.

To identify novel mosquito miRNAs, we used a combination of miRDeep2 [23] and randfold [24] to ask whether non-annotated sequences mapping to the mosquito genomes demonstrated folding properties of pre-miRNAs hairpins. Each novel miRNA follows both expression and biogenesis criteria set forth for identifying new miRNAs, which include (i) a small RNA of appropriate and discrete length (19–24 nt), (ii) arising from one arm of a hairpin precursor, (iii) presence of the star strand, and (iv) evolutionary conservation [5,25]. The software RNA-fold was used to predict and analyze the structure of new miRNAs [13,15].

### Northern blot

Sample collections from different developmental stages of *An. anthropophagus* for northern blots, are briefly described below. Embryo collections were made at 0–24 hours after placing a damp collection cup within a cage. In the larval sample collection, we did not separate early and late larval samples and used one mixed larval sample (I-IV instars) instead. Pupa samples were collected from a pool of varied ages. Adults one to five days following eclosion were collected.

All samples were either directly processed for RNA isolation or flash frozen on liquid nitrogen immediately following collection, then stored at −80°C. The isolation method of total RNA is unbiased for each sample as described above. The quantity of total RNA used for each sample is ~ 20 μg. Northern blots were carried out according to the previous reports [13]. Briefly, total RNA were loaded onto 15% denaturing polyacrylamide gels, and run beside 19 and 23 nucleotide long ssDNA markers. The RNA gels were transferred to Bright- Star-Plus nylon membranes (Ambion), crosslinked using a UV crosslinker, and prehybridized, then hybridized overnight in the ULTRAhyb-Oligo Hybridization Buffer (Ambion) with the appropriate DIG-labeled probe at 42°C. Wash conditions were the same as described before [13]. Antisense 5′ digoxigenin-labelled miRCURY LNA probes were prepared by Exiqon (Vedbaek, Denmark). Probe sequences were as follow, aan-miR-184: CCCTTATCAGTTCTCCGTCCA; aan-miR-989: GTACCACTACGTCACATCACA; aan-miR-998: GAGCTGAATCTCATG GTGCTA; aan-miR-2943: TTTGCCTGCAAGTGCCTACTTAA; aan-miR-1000: ACTG CTGTGTCAGGCAATAT.

## Results and discussion

### High-throughput sequencing of small RNAs

**Figure 1:**
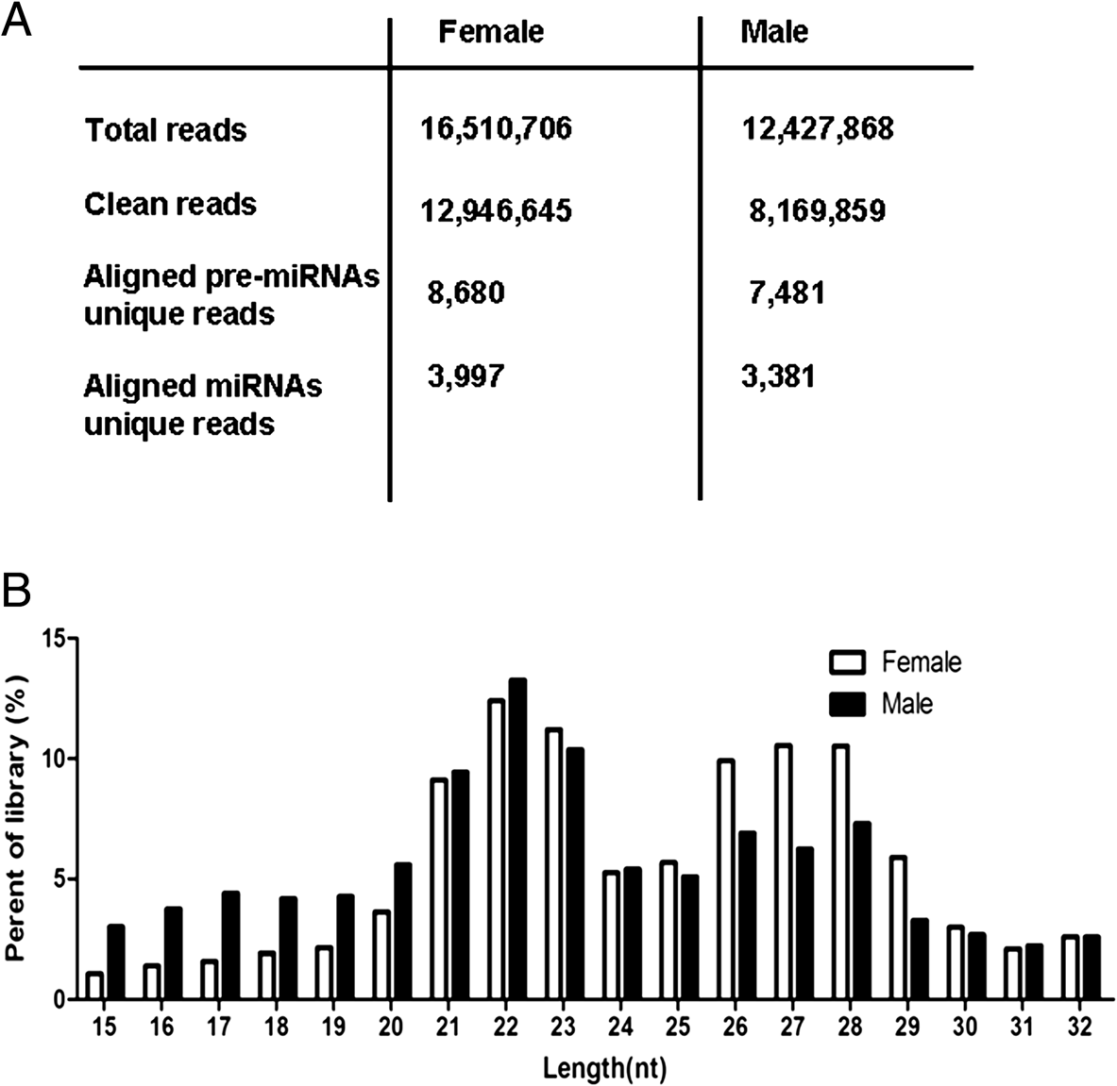
**Size distributions of small RNA from female and male *Anopheles anthropophagus *adults. A)** Breakdown of the total number of reads obtained for each library. The number of reads mapping to pre-miRNA and mature miRNA strands is reported. **B)** The comparison of small RNA libraries (15–32 nt) from female and male adults.

In order to identify miRNAs from the *An. anthropophagus* mosquito, we isolated small RNAs from male and female adults separately. Small RNA libraries were subjected to Illumina Hiseq2000- based high-throughput sequencing, 12.43 million and 16.51 million raw reads were yielded from the total RNA of male and female adults separately. After filtering for linker sequences, and removing ambiguous reads, high quality clean reads with sizes ranging from 15 to 32 nt were collected, of which there were 8,169,859 (65.74%) reads for male, 12,946,645 (78.41%) reads for female (Figure 1A). Among the clean reads, the majority (32.75% for female, 33.07% for male) ranged from 21 to 23 nt in length (Figure 1B).

There is also an elevated population of small RNAs 25–30 nt in length, which may represent piwi-interacting RNAs (piRNAs). piRNAs are known to derive from a Dicer-independent manner from single-stranded precursors and suppress repetitive sequences including transposable elements [26,27]. Recently piRNAs have been shown to be involved in the innate immunity of mosquitoes [26,27]. Further investigation of the piRNA pathway in *An. anthropophagus* mosquitoes will improve our understanding of how this important vector species may defend against pathogens.

### Most miRNAs are orthologs of known mosquito miRNAs

The miRNA sequencing reads from male and female adults of *An. anthropophagus* mosquitoes were aligned to the known miRNA strands present in miRBase (version 20.0). For the pre-miRNAs and mature miRNA library, 8,169,859 clean reads from the male adult library corresponded to 7,481 pre-miRNAs (0.55%) and 3,381 mature miRNAs (0.25%); 12,946,645 clean reads from the female adult library corresponded to 8,680 pre-miRNAs (0.4%) and 3,997 mature miRNAs (0.18%) (Figure 1A).

*An. anthropophagus*, *An. gambiae* and *Ae. Aegypti* are important mosquito-borne disease vectors in tropical and sub-tropical areas*. An. gambiae and An. anthropophagus* transmit plasmodium and cause malaria, and *Ae. Aegypti* is the primary vector for yellow fever and dengue fever [13,18,28]. The genome sequences of *An. gambiae* and *Ae. Aegypti* have been published [13,18,20]. However, *An. anthropophagus* genome is not yet sequenced. Since miRNA sequences are highly conserved between species [13,15], we aligned sequencing reads to known mature miRNAs and the pre-miRNA library of *An. gambiae* (aga) and *Ae. aegypti* (aae). 81 known miRNA expressions were detected in *An. anthropophagus* (Additional file 1: Table S1), of which 62 miRNAs strands mapped with 100% identity to the library of *An. gambiae*, and 73 miRNAs strands mapped with 100% identity to the library of *Ae. aegypti*. 53 miRNAs strands were found to be consistent in these three different mosquito species. Normally, some miRNAs can be found both at 5p and 3p of the same pre-miRNAs [29]. So, the locations of *An. anthropophagus* miRNAs were subsequently analyzed. We identified 14 miRNAs strands, which located both at of the 5p and 3p of the same pre-miRNAs. However, the miRNAs -3p were only found in the library of *Ae. Aegypti.* Therefore, these 14 miRNAs and the miRNAs only found in the library of *Ae. Aegypti* were located according to the genome of *Ae. Aegypti,* (Additional file 1: Table S1). The other miRNAs were located according to the genome of *An. gambiae* (Additional file 1: Table S1).

### Identification of novel mosquito miRNAs

**Figure 2:**
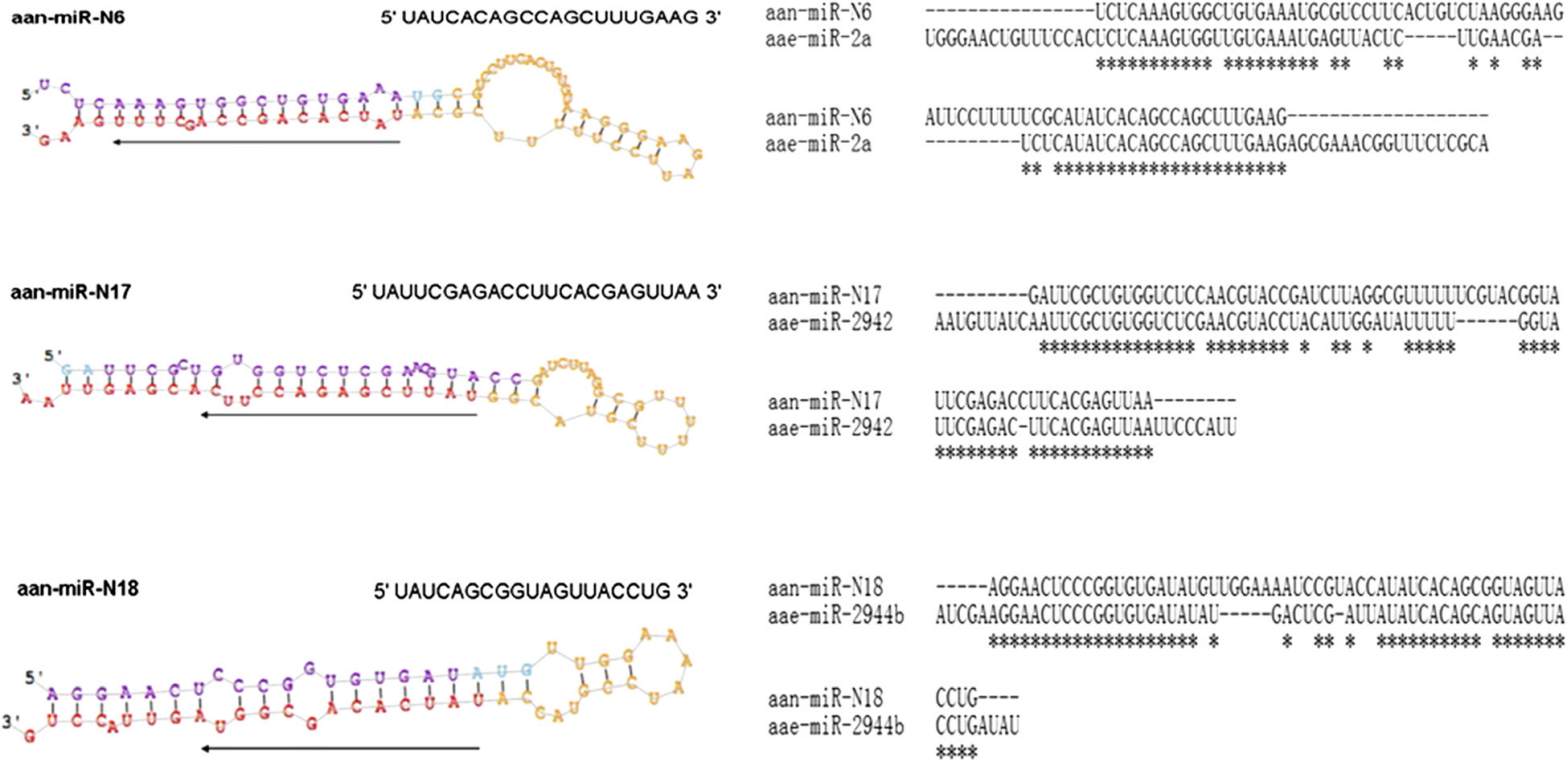
**Alignments and stem-loop structures of three novel mosquito pre-miRNAs.** See Table 2 for naming and sequence locations of these miRNAs. Left panels are the hairpin structures. Right panels are the sequence alignments between *An. anthropophagus* miRNAs (aan-miR) and *Ae. Aegypti* miRNAs (aae-miR). Arrows point to the mature miRNA sequences from 5′ to 3′.

Except for 81 known miRNAs*,* an additional 21 novel miRNAs have only been found in *An. anthropophagus* mosquitoes (Table 1). All 21 mature miRNAs have multiple hits from small RNA sequencing, and each miRNA arises from RNA structures which fold into canonical pre-miRNA hairpins (Figure 2 and Additional file 2: Figure S1) confirming their status as miRNAs. Eight of the new miRNAs reside on the 5p arms of their respective precursors, while the remaining 13 miRNAs reside on the 3p arms. There are no miRNAs found both at 5p and 3p of the same pre-miRNAs (Figure 2 and Additional file 2: Figure S1).

**Table 1:** **The novel microRNAs (miRNAs) identified from *An. anthropophagus *** **adults**

**Name**^**a**^	**Location at genome**^**b**^	**Sequence**^**c**^	**Length**	**Location**^**d**^
aan-miR-N1 ^#^	137818-137890	CGCUGCAGUACUGGCGCC	18	5p
aan-miR-N2 ^#^	275878-275942	AUCCGGUGAUAGGCUGACCCG	21	3p
aan-miR-N3 ^#^	576224-576298	UUAGAAUGUGGAAUCUGUUU	20	5p
aan-miR-N4 ^#^	120072-120133	UUGGUGUUAUAUCUUACAGUGAG	23	3p
aan-miR-N5 ^#^	748862-748925	UUGGUGUUAUAUCUUACAGUGAG	23	3p
aan-miR-N6	37757410-37757490	UAUCACAGCCAGCUUUGAAG	20	3p
aan-miR-N7	7197873-7197941	UGCAUUCAGUGGGGCGGUCGUG	22	3p
aan-miR-N8	8265177-8265239	UGUUAACUGUAAGACUGUGUCG	22	3p
aan-miR-N9	13042402-13042472	UAGCACCAUGAGAUUCAGCUC	21	3p
aan-miR-N10	15199831-15199898	UCAAUUCCGUAGUGCAUUGCAGU	22	5p
aan-miR-N11	15821040-15821118	UUGGUGUUAUAUCUUACAGUGAG	22	3p
aan-miR-N12	22427284-22427343	GUAGGCCGGCGGAAACUACUUGC	22	3p
aan-miR-N13	25664558-25664612	UUGGCCGGUACGGGCUGACCGGGC	23	5p
aan-miR-N14	44246727-44246783	UGAACCGGCGUAGCGUGAAAGCA	22	5p
aan-miR-N15	52693314-52693371	CUAAGUACUAGUGCCGCAGGAG	21	5p
aan-miR-N16	17216221-17216288	UUAGAAUGUGGAAUCUGUUU	19	5p
aan-miR-N17	39686860-39686930	UAUUCGAGACCUUCACGAGUUAA	22	3p
aan-miR-N18	43009064-43009125	UAUCAGCGGUAGUUACCUG	18	3p
aan-miR-N19	1256011-1256078	GUGCAUUGUAGUUGCAUUGCA	20	3p
aan-miR-N20	13576281-13576333	GUUGCUGUCCGCUGAAGCA	18	3p
aan-miR-N21	14375922-14375980	UGGCAAGAUGUUGGCAUAGCAGCU	23	5p

Note: ^a^the provisional name of miRNA, the abbreviation of Novel “N” was added to make a differentiation with know miRNA in Additional file 1: Table S1; ^b^the start, and end positions refer to the locations of the pre-miRNA hairpins at the reference genome of *Anopheles gambiae*, or ^#^*Aedes aegypti*; ^c^sequence of mature miRNA; ^d^location of a mature miRNA at the 5p or 3p arm of its precursor.

Shown in Figure 2 are the sequence alignments of 3 novel miRNAs sequences discovered in this study and the hairpins they form. Sequence blast analysis suggests that miR-N6, miR-N17 and miR-N18 are separately found to be orthologous genes with miR-2a, miR-2942 and miR-2944b in *Ae. Aegypti* (Figure 2). miR-N4, −N5, −N8, −N9, −N10, −N11, −N12 and miR-N15 are found the orthologous genes in *Drosophila melanogaster* or *Bombyx mori*. It is suggested that most miRNAs are relatively conserved in arthropods [25]. miR-N19 is found to be the orthologous gene with miR-33, which is a family of microRNA precursors in several animal species, including humans [30]. Unexpectedly, miR-N21 is found to be the orthologous gene with miR-31, which has been characterised as a tumour suppressor miRNA, with its levels varying in breast cancer cells according to the metastatic state of the tumor [31,32]. No matches were found for miR-N7.

### miRNAs show sexual difference expression in adults

The miRNA expression levels in male and female adults, based on the number of reads obtained, varied greatly. For the same miRNAs, the expression level in male and female adults shows a significant difference. Especially, 29 miRNAs show sexual difference expression: >2-fold in the read count of the same miRNA in male and females (Table 2). For example, miR-100, −1000, −125, −193, −137, and −124 in male adults shows an increase in expression count over 2 times that in female adults. But miR-1175-3p, −989, −9b and -9c in male adult shows a decrease in expression count over 2 times that in female adults. Especially for miR-989, which has only 2 read counts in the male adult but 1,324 read counts in the female adult, the expression level in females is more than 300 times that in males. Li *et al*. (2009) also found that miR-989 has only 2 read counts in the embryo stage, but has 33 read counts in the sugar fed midgut of female adults, indicating that miR-989 may be involved in the physiological activity of female mosquitoes [13]. Meanwhile, miR-281, −1174, and −1175 were only found to be expressed in the midgut of adults [13,15]. miRNAs are essential for the regulation of the complex physiological activity of mosquitoes, allowing them to respond to environmental and developmental signals [13,29]. Thus, the miRNAs identified in our study provided novel resources for better understanding of the biology of *An. anthropophagus*.

**Table 2:** **Different expression levels of microRNAs (miRNAs) identified from the male and female adults of *An. anthropophagus***

**Name**	**The read count of male adult**^**a**^	**The read count of female adult**^**b**^	**The revised read count of male adult**^**c**^	**The ratio of female/male**^**d**^
aan-miR-100	1361	1199	2612	0.46
aan-miR-1000	92	84	177	0.48
aan-miR-1175	366	1441	702	2.05
aan-miR-1175-3p	344	1439	660	2.18
aan-miR-124	9	3	17	0.17
aan-miR-125	74	68	142	0.48
aan-miR-125-3p	98	68	188	0.36
aan-miR-137	22	15	42	0.36
aan-miR-193	17	12	33	0.37
aan-miR-263b	531	398	1019	0.39
aan-miR-263b-3p	3	0	6	―
aan-miR-276-3p	9	3	17	0.17
aan-miR-277-3p	339	246	651	0.38
aan-miR-282	11	10	21	0.47
aan-miR-2944a	65	48	125	0.38
aan-miR-2944b	6	1	12	0.09
aan-miR-307	4	18	8	2.34
aan-miR-309a	4	1	8	0.13
aan-miR-315	4	24	8	3.13
aan-miR-7	5	4	10	0.42
aan-miR-79	0	8	0	―
aan-miR-79-3p	1	4	2	2.08
aan-miR-929	0	2	0	―
aan-miR-981	12	6	23	0.26
aan-miR-988-3p	0	6	0	―
aan-miR-989	2	1324	4	344.92
aan-miR-9b	118	607	226	2.68
aan-miR-9c	535	2424	1027	2.36
aan-miR-9c-3p	47	214	90	2.37

Note: ^a^the number of sequence read count in the male adult miRNAs libraries obtained by illumina Hiseq2000 sequencing; ^b^the number of sequence read count in the female adult miRNAs libraries obtained by illumina Hiseq2000 sequencing. The total reads of male adult is 281,733, the total reads of female adult is 540,720; ^c^the revised reads of male adult = the male adult reads × (540720÷281733); ^d^the ratio of female/male = the read count of female÷the revised read count of male.

### Confirmation of mosquito miRNAs

**Figure 3:**
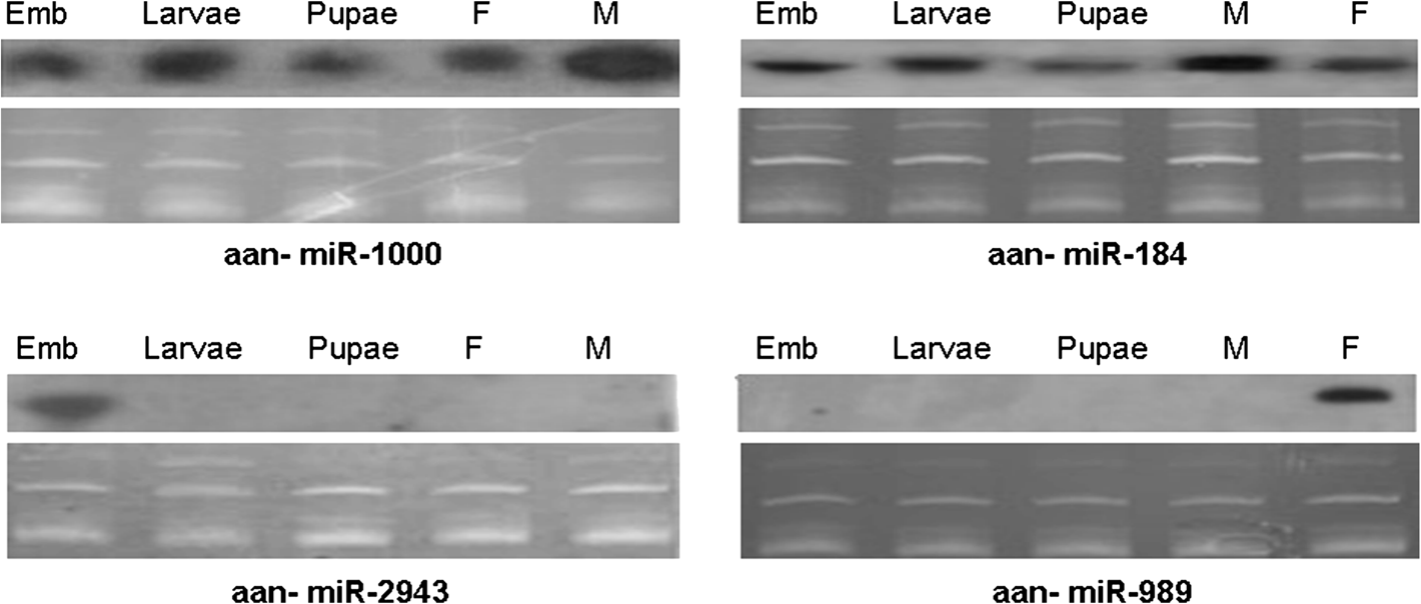
**Expression patterns of four mosquito-specific miRNAs in *An. anthropophagus *are homologs of previously known miRNAs.** Only *An. anthropophagus* RNA samples were examined. The top panels are northern results and the bottom panels are RNA gel images for verification of small ribosomal RNA and tRNA integrity and loading of total RNA. Emb, pooled embryos between 0–36 hr after egg deposition; Larvae; mixed instar larvae; Pupae, mixed pupae; F, adult females one to five days after emergence; M, adult males one to five days after emergence. About 20 μg of total RNA were used per sample.

In order to confirm the small RNA sequencing results, and to determine the expression patterns of these miRNAs in different developmental stages, we chose three conserved miRNAs: miR-184, −989 and miR-1000 represented in our sequencing data for further northern blot analysis. miR-2943, which has been proved to be only expressed in the embryonic stage, was taken as the positive control. All four miRNAs showed signals at ~20 nt by northern blot during at least one of the developmental stages. The expression patterns of four conserved miRNAs are shown in Figure 3. The patterns of miR-184, −2943 and miR-1000 are similar to the patterns found in *Ae. Aegypti*[13]. miR-184 and miR-1000 are present in *An. anthropophagus* embryo, larvae, pupa, and adult stages. The expression level of these two miRNAs in male adults is higher than those in female adults by comparing with the blot signal, which is consistent with the sequencing data. miR-2943 is only detected in the embryonic stage. As described above, the expression count of miR-989 in female adults is signally abundant than that in male adult by sequencing, and miR-989 was only detected in the female adults, there was no signal in embryo, larvae, pupa and male stages by northern blot.

### Potential impact of trizol in the loss of low GC miRNAs

Trizol is commonly used for RNA isolation [15,29,33]. Kim *et al*. (2012) found that the structured small RNAs with low GC content are recovered inefficiently when a small number of cells (<500,000) are used for RNA isolation with trizol [34]. Since small RNA needs to base pair with other longer RNA for effective precipitation, it is plausible that the higher concentration of starting material contains more large RNA and is helpful for precipitating the small RNA [26]. In our study, 100 male or female mosquitoes were collected for the RNA isolation, and the quantities of total RNA were ~ 111 μg for male and ~ 159 μg for female. In addition, the quantities of total RNA for egg, larvae and pupae were also more than 100 μg in the trizol extraction. These high quantities of total RNA will help to prevent the small RNA for being lost in the isolation with trizol. Meanwhile, the GC content of aan-miR-1000、-184、-989 and aan-miR-2943 in our study were compared with the low GC miRNA (hsa-miR-141 and miR-200c) in Kim’s paper [34]. As shown in Additional file 3: Table S2, the GC contents of aan-miR-1000 and miR-2943 are very contiguous to miR-141 and miR-200c, which is about 43.5%. So, aan-miR-1000 and miR-2943 also belong to low GC miRNAs. The aan-miR-1000 isolated by trizol can be found in both male and female adults in the sequencing experiments, which further demonstrated that the trizol-related impact on low GC miRNA isolation in our study has been limited in sequencing experiments.

In order to certify that the potential of trizol bias issues were also avoided in Northern experiments, the isolation of RNA from new batches of eggs, larvae, pupae and adult mosquitoes (female and male) were repeated with mirVana miRNA isolation kit (Ambion, USA) rather than trizol. The Northern blot results of miR-1000, −184, −2943 and miR-989 with mirVana miRNA isolation kit are similar to the patterns with trizol isolation (Additional file 4: Figure S2). As described before, miR-1000 and miR-2943 belong to the low GC miRNAs family. miR-1000 is present in embryonic, larval, pupal, and adult stages, and the expression level in male adults is higher than those in female adults by comparing with the blot signal. In contrast, miR-2943 is only present in the embryonic stage. The Northern blot results indicated that the similar expression patterns of the low GC miRNAs were detected both in mirVana miRNA kit isolation and trizol method, and the potential trizol bias issues were overcome in the Northern experiments.

## Conclusion

In the present study, the miRNA expression profile in *An. anthropophagus* were investigated and 102 miRNAs including 81 known and 21 new miRNAs were identified from *An. anthropophagus*. Furthermore, we also investigated the expression difference of miRNAs in male and female adults as well as different developmental stages, indicating several miRNAs exhibited sexual difference and stage-specific differences. Although the loss of low GC miRNAs was overcome in this study, we should pay attention to the trizol bias issue in future experiments. The present study represented the first global characterization of *An. anthropophagus* miRNAs, which provides novel resources for better understanding of the biology of the mosquitoes, which, in turn, has implications for the effective control of mosquito-borne infectious diseases.

## Competing interests

The authors declare that they have no competing interests.

## Authors’ contributions

SHL and WQL conceived and designed the study. WQL, HCH CCX and CXL performed the experiments, analyzed the data and drafted the manuscript. SHL and FT critically revised the manuscript. All authors read and approved the final manuscript.

## Supplementary Material

Additional file 1: Table S1 Known microRNAs (miRNAs) identified from *An. anthropophagus* adult. Click here for file

Additional file 2: Figure S1 The stem-loop structure of novel mosquito pre-miRNAs. Click here for file

Additional file 3: Table S2 The GC content of the microRNA (miRNA) in Northern blot. Click here for file

Additional file 4: Figure S2 Expression patterns of four mosquito-specific miRNAs isolated with mirVana miRNA isolation kit. Click here for file
