# Supplementary material for: Identification and characterization of the expression profile of microRNAs in Anopheles anthropophagus
Source: Parasit Vectors. 2014 Apr 1;7:159. doi: 10.1186/1756-3305-7-159 (PMC4022070; doi:10.1186/1756-3305-7-159)
Supplement: Additional file 1: Table S1 — Known microRNAs (miRNAs) identified from An. anthropophagus adult. [file 1756-3305-7-159-S1.doc]

**Table S1 Known microRNAs (miRNAs) identified from *Anopheles anthropophagus* adult**

| **Name** | **Location at genome**^a^  **[strand]** | **aga**^b^ | **aae**^c^ | **Location**^d^ | **Sequence**^e^ | **Length** |
| --- | --- | --- | --- | --- | --- | --- |
| aan-bantam | 33274497-33274608  [+] | Y | Y | 5p | UGAGAUCACUUUGAAAGCUGAUU | 23 |
| aan-let-7 | 10270707-10270797  [-] | Y | Y | 5p | UGAGGUAGUUGGUUGUAUAGU | 21 |
| aan-miR-1 | 10569153-10569251  [-] | Y | Y | 3p | UGGAAUGUAAAGAAGUAUGGAG | 22 |
| aan-miR-10 | 59711764-59711866  [-] | Y | Y | 5p | ACCCUGUAGAUCCGAAUUUGUU | 22 |
| aan-miR-100 | 10274677-10274768  [-] | Y | Y | 5p | AACCCGUAGAUCCGAACUUGUG | 22 |
| aan-miR-1000 | 13241764-13241857  [+] | Y | Y | 5p | AUAUUGUCCUGUCACAGCAGU | 21 |
| aan-miR-11 | 13042021-13042111  [+] | Y | Y | 3p | CAUCACAGUCUGAGUUCUUGCU | 22 |
| aan-miR-1174 | 9129498-9129675  [+] | Y | Y | 3p | UCAGAUCUACUUAAUACCCAU | 21 |
| aan-miR-1175^#^ | 1648025-1648108  [+] | Y | Y | 5p/3p | AAGUGGAGUAGUGGUCUCAUCG/UGAGAUUCUACUUCUCCGACUUAA | 22/24 |
| aan-miR-12 | 37888036-37888153  [-] | Y | Y | 5p | UGAGUAUUACAUCAGGUACUGGU | 23 |
| aan-miR-124 | 29001979-29002062  [+] | Y | Y | 3p | UAAGGCACGCGGUGAAUGCCAAG | 23 |
| aan-miR-125 | 10270159-10270271  [-] | Y | Y | 5p | UCCCUGAGACCCUAACUUGUGA | 22 |
| aan-miR-13^#^ | 888735-888819  [-] | ? | Y | 3p | UAUCACAGCCAUUUUGACGAGUU | 23 |
| aan-miR-133 | 15075186-15075263  [-] | Y | Y | 5p | UUGGUCCCCUUCAACCAGCUGU | 22 |
| aan-miR-137 | 11039425-11039516  [+] | Y | Y | 3p | UAUUGCUUGAGAAUACACGUAG | 22 |
| aan-miR-13b | 37757563-37757657  [-] | Y | ? | 3p | UAUCACAGCCAUUUUGACGAGU | 22 |
| aan-miR-14 | 24898040-24898134  [+] | Y | Y | 3p | UCAGUCUUUUUCUCUCUCCUAU | 22 |
| aan-miR-184 | 42632356-42632438  [+] | Y | Y | 3p | UGGACGGAGAACUGAUAAGGGC | 22 |
| aan-miR-1890 | 21181081-21181177  [-] | Y | Y | 3p | UGAAAUCUUUGAUUAGGUCUGG | 22 |
| aan-miR-1891 | 5819037-5819138  [-] | Y | Y | 5p | UGAGGAGUUAAUUUGCGUGUUU | 22 |
| aan-miR-190 | 46755771-46755872  [-] | Y | Y | 5p | AGAUAUGUUUGAUAUUCUUGGUUG | 24 |
| aan-miR-193^#^ | 717065-717189  [+] | ? | Y | 3p | UACUGGCCUACUAAGUCCCAAC | 22 |
| aan-miR-2 | 37757102-37757189  [-] | Y | ? | 3p | UAUCACAGCCAGCUUUGAUGAGC | 23 |
| aan-miR-210 | 21450334-21450414  [+] | Y | Y | 3p | CUUGUGCGUGUGACAACGG | 19 |
| aan-miR-219 | 21839671-21839758  [-] | Y | Y | 5p | UGAUUGUCCAAACGCAAUUCUUG | 23 |
| aan-miR-252^#^ | 1580020-1580091  [-] | ? | Y | 5p/3p | UAAGUACUAGUGCCGCAGGAG/CUGCUGCCCAAGUGCUUAUCG | 21/21 |
| aan-miR-263a | 12395475-12395561  [+] | Y | Y | 5p | AAUGGCACUGGAAGAAUUCACGG | 23 |
| aan-miR-263b^#^ | 2323832-2323922  [+] | Y | Y | 5p/3p | CUUGGCACUGGGAGAAUUCACAG/UGGAUCUUUUCGUGCCAUCGU | 23/21 |
| aan-miR-275 | 38377341-38377436  [+] | Y | Y | 3p | UCAGGUACCUGAAGUAGCGC | 20 |
| aan-miR-276 | 18991753-18991845  [+] | Y | Y | 5p/3p | AGCGAGGUAUAGAGUUCCUA/UAGGAACUUCAUACCGUGCUCU | 20/22 |
| aan-miR-2765^#^ | 5248293-5248380  [+] | ? | Y | 5p | UGGUAACUCCACCACCGUUGGC | 22 |
| aan-miR-277^#^ | 508799-508893  [+] | Y | Y | 5p/3p | UAAAUGCACUAUCUGGUACGAC/CGUGUCAGAAGUGCAUUUACA | 22/21 |
| aan-miR-278 | 34112620-34112703  [-] | Y | Y | 3p | ACGGACGAUAGUCUUCAGCGGCC | 23 |
| aan-miR-279 | 55573326-55573415  [-] | Y | Y | 3p | UGACUAGAUCCACACUCAUUAA | 22 |
| aan-miR-281^#^ | 134442-134538  [+] | Y | Y | 5p/3p | UGUCAUGGAAUUGCUCUCUUUA/UGUCAUGGAAUUGCUCUCUUUA | 22/22 |
| aan-miR-282^#^ | 992964-993098  [+] | ? | Y | 5p | AAGAGAGCUAUCCGUCGAC | 19 |
| aan-miR-283 | 37890033-37890125  [-] | Y | Y | 5p | CAAUAUCAGCUGGUAAUUCUGGGC | 24 |
| aan-miR-285^#^ | 3339126-3339230  [-] | ? | Y | 3p | UAGCACCAUUCGAAAUCAGUAC | 22 |
| aan-miR-286 | 43009394-43009490  [-] | Y | ? | 3p | UGACUAGACCGAACACUCGCGUC | 23 |
| aan-miR-286b^#^ | 907326-907422  [+] | ? | Y | 3p | UGACUAGACCGAACACUCGUAUCCC | 25 |
| aan-miR-2944a^#^ | 825449-825511  [+] | ? | Y | 5p | GAAGGAACUUCUGCUGUGAUCUGA | 24 |
| aan-miR-2944b^#^ | 825312-825374  [+] | ? | Y | 5p | GAAGGAACUCCCGGUGUGAUAUA | 23 |
| aan-miR-2945^#^ | 958686-958768  [+] | ? | Y | 3p | UGACUAGAGGCAGACUCGUUUA | 22 |
| aan-miR-2a^#^ | 888581-888677  [-] | ? | Y | 3p | UAUCACAGCCAGCUUUGAAGAGC | 23 |
| aan-miR-2b^#^ | 887244-887330  [-] | ? | Y | 3p | UAUCACAGCCAGCUUUGAUGAGCU | 24 |
| aan-miR-2c^#^ | 889085-889162  [-] | ? | Y | 3p | UAUCACAGCCAGCUUUGAUGAGC | 23 |
| aan-miR-305^#^ | 495587-495674  [+] | Y | Y | 5p/3p | AUUGUACUUCAUCAGGUGCUCUGG/C  GGCACAUGUUGGAGUACACUUA | 24/23 |
| aan-miR-306 | 5888599-5888675  [-] | Y | ? | 5p | UCAGGUACUGGAUGACUCUCAG | 22 |
| aan-miR-307 | 34537911-34538009  [+] | Y | Y | 3p | UCACAACCUCCUUGAGUGAG | 20 |
| aan-miR-308 | 508970-509045  [+] | Y | Y | 5p/3p | AAUCACAGGAGUAUACUGUGAG/AAUCACAGGAGUAUACUG | 22/18 |
| aan-miR-309 | 43008710-43008784  [-] | Y | ? | 3p | UCACUGGGCAAAGUUUGUCGCA | 22 |
| aan-miR-309a-1^#^ | 907874-907965  [+] | ? | Y | 3p | UCACUGGGCAAAGUUUGUCGC | 21 |
| aan-miR-31^#^ | 483181-483329  [-] | ? | Y | 5p | UGGCAAGAUGUUGGCAUAGCUGA | 23 |
| aan-miR-315^#^ | 104128-104219  [+] | Y | Y | 5p/3p | UUUUGAUUGUUGCUCAGAAAGC/CUUUCGAGCAGUAAUCAAAGUC | 22/22 |
| aan-miR-317 | 28251997-28252086  [-] | Y | Y | 3p | UGAACACAUCUGGUGGUAUCUCAG | 24 |
| aan-miR-34 | 28232667-28232756  [-] | Y | Y | 5p | UGGCAGUGUGGUUAGCUGGU | 20 |
| aan-miR-375-1 | 51640560-51640668  [-] | Y | Y | 3p | UUUGUUCGUUUGGCUCGAGUUA | 22 |
| aan-miR-7 | 8302899-8302985  [-] | Y | Y | 5p | UGGAAGACUAGUGAUUUUGUUGU | 23 |
| aan-miR-71^#^ | 889378-889454  [-] | ? | Y | 5p/3p | AGAAAGACAUGGGUAGUGAGAU/UCUCACUACCUUGUCUUUCAUG | 22/22 |
| aan-miR-79^#^ | 213262-213357  [+] | Y | Y | 5p/3p | UAAAGCUAGAUUACCAAAGCAU/UAAAGCUAGAUUACCAAAGCAU | 22/22 |
| aan-miR-8 | 876043-876121  [+] | Y | Y | 5p/3p | UAAUACUGUCAGGUAAAGAUGUC/CAUCUUACCGGGCAGCAUUAGA | 23 |
| aan-miR-87 | 261171-261271  [-] | Y | ? | 3p | GGUGAGCAAAUAUUCAGGUGU | 21 |
| aan-miR-927 | 18737367-18737460  [+] | Y | ? | 5p | UUUAGAAUUCCUACGCUUUACC | 22 |
| aan-miR-929 | 38819434-38819522  [-] | Y | ? | 3p | CUCCCUAACGGAGUCAGAUUG | 21 |
| aan-miR-92a | 39181114-39181203  [+] | Y | Y | 3p | UAUUGCACUUGUCCCGGCCUA | 21 |
| aan-miR-92b | 39200607-39200694  [+] | Y | Y | 3p | AAUUGCACUUGUCCCGGCCUGC | 22 |
| aan-miR-932-3p^#^ | 154097-154192  [-] | ? | Y | 3p | UCAAUUCCGUAGUGCAUUGCAG | 22 |
| aan-miR-957 | 14911227-14911321  [+] | Y | Y | 3p | UGAAACCGUCCAAAACUGAGGC | 22 |
| aan-miR-965-1 | 11096169-11096272  [+] | Y | ? | 3p | UAAGCGUAUAGCUUUUCCCAUU | 22 |
| aan-miR-970 | 112021-112119  [-] | Y | Y | 3p | UCAUAAGACACACGCGGCUAU | 21 |
| aan-miR-980^#^ | 1043019-1043095  [+] | ? | Y | 3p | UAGCUGCCUAGUGAAGGGC | 19 |
| aan-miR-981 | 1228286-1228381  [+] | Y | Y | 3p | UUCGUUGUCGACGAAACCUGCA | 22 |
| aan-miR-988^#^ | 623050-623124  [-] | Y | Y | 5p/3p | CCCCUUGUUGCAAACCUCACGC/GUGUGCUUUGUGACAAUGAGA | 22/21 |
| aan-miR-989 | 2905394-2905525  [+] | Y | Y | 3p | UGUGAUGUGACGUAGUGGUAC | 21 |
| aan-miR-993 | 59284603-59284723  [-] | Y | Y | 3p | GAAGCUCGUUUCUAUAGAGGUAUCU | 22 |
| aan-miR-996 | 55572841-55572910  [-] | Y | Y | 3p | UGACUAGAUUACAUGCUCGUC | 21 |
| aan-miR-999^#^ | 2315099-2315178  [+] | ? | Y | 3p | UGUUAACUGUAAGACUGUGUCU | 22 |
| aan-miR-9a | 15089289-15089370  [-] | Y | Y | 5p | UCUUUGGUUAUCUAGCUGUAUGA | 23 |
| aan-miR-9b | 5887785-5887878  [-] | Y | Y | 5p | UCUUUGGUGAUUUUAGCUGUAUGC | 24 |
| aan-miR-9c^#^ | 186226-186308  [+] | Y | Y | 5p/3p | UCUUUGGUAUUCUAGCUGUAGA/UAAAGCUUUAGUACCAGAGGUC | 22/22 |
| aan-miR-iab-4 | 60297556-60297639 [+] | Y | Y | 5p | ACGUAUACUGAAUGUAUCCUGA | 22 |

Note: ^a^ the start, and end positions refer to the locations of the pre-miRNA hairpins at the reference genome of *Anopheles gambiae*, or  ^#^ *Aedes aegypti*; ^b^ *Anopheles gambiae* (*A. gambiae*); ^c^ *Aedes aegypti* (*A. aegypti*); ^d^ location of a mature miRNA at the 5p or 3p arm of its precursor; ^e^ sequence of mature miRNA
