# Supplementary material for: Identification and characterization of the expression profile of microRNAs in Anopheles anthropophagus
Source: Parasit Vectors. 2014 Apr 1;7:159. doi: 10.1186/1756-3305-7-159 (PMC4022070; doi:10.1186/1756-3305-7-159)
Supplement: Additional file 2: Figure S1 — The stem-loop structure of novel mosquito pre-miRNAs. [file 1756-3305-7-159-S2.doc]

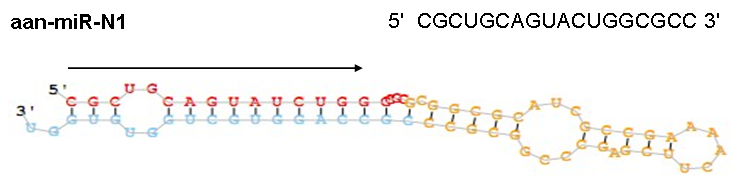


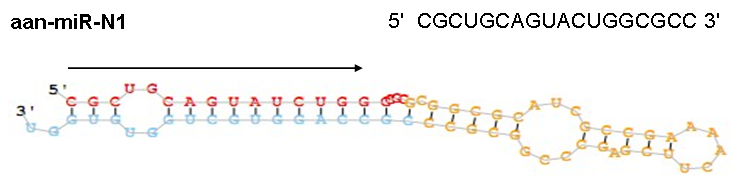


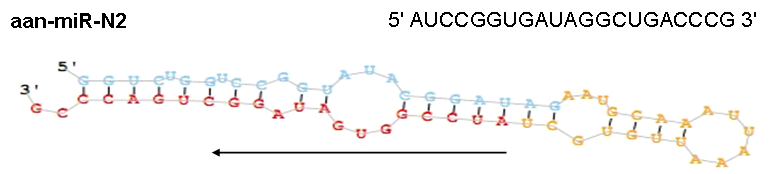


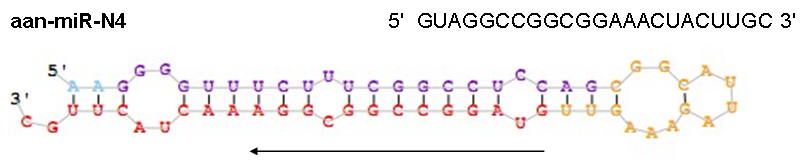


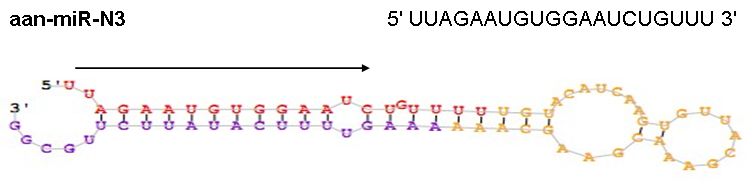


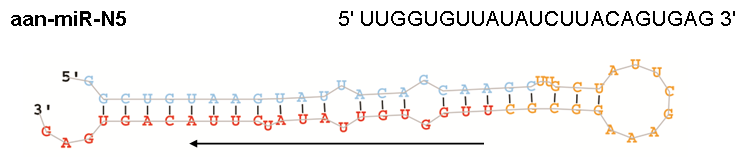


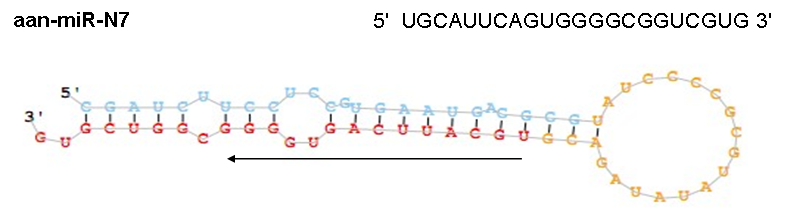


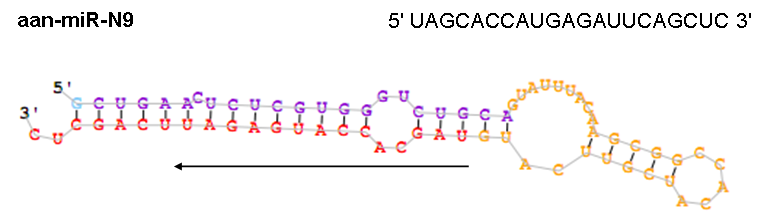


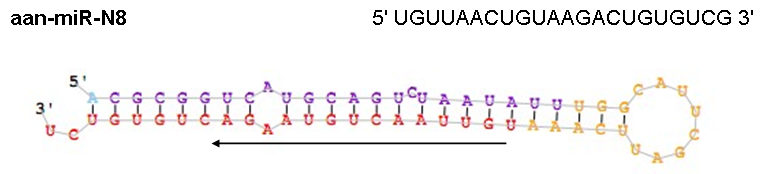


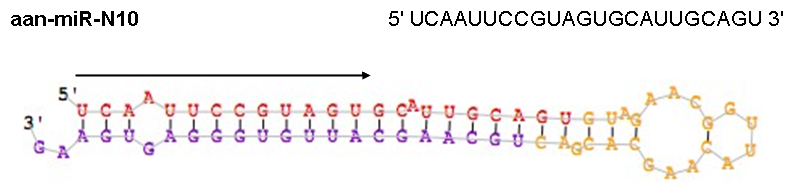


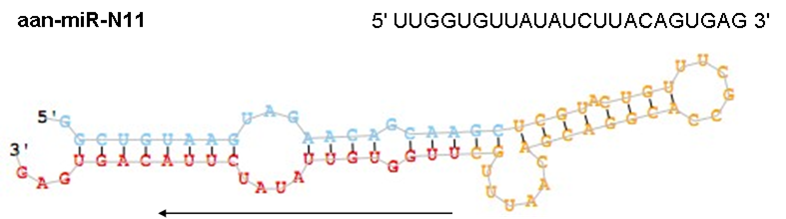


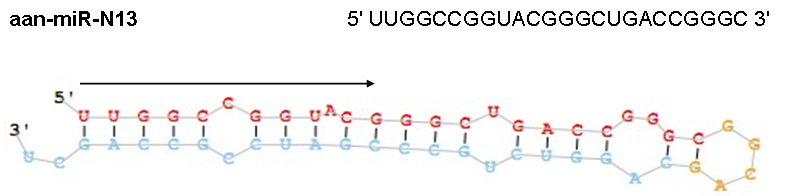


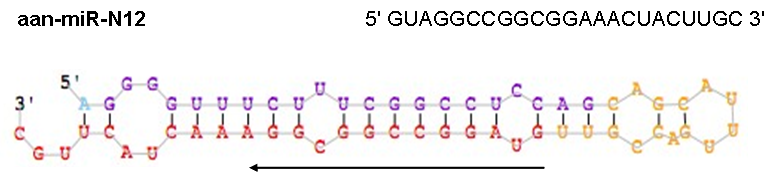


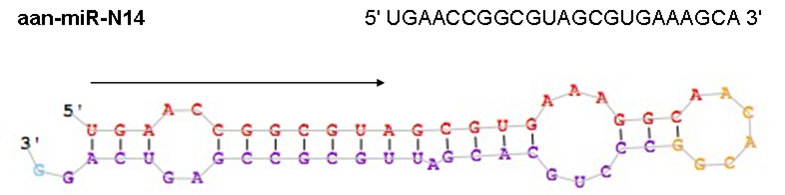


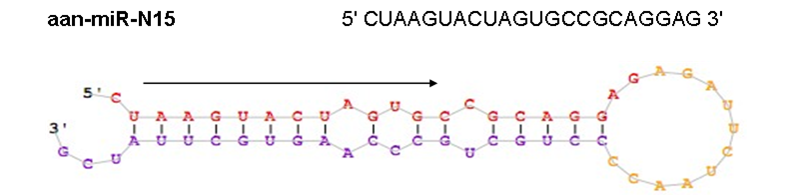


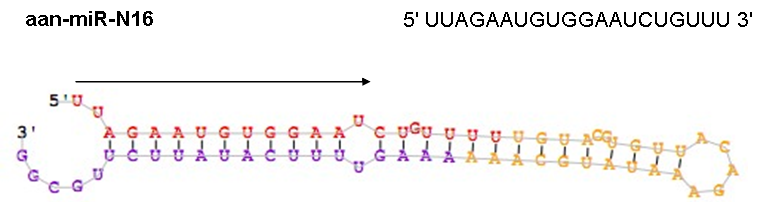


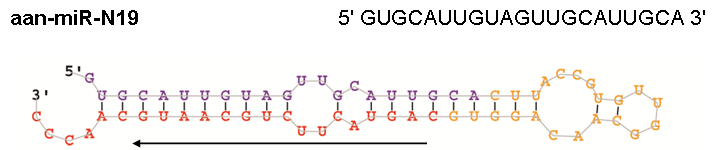


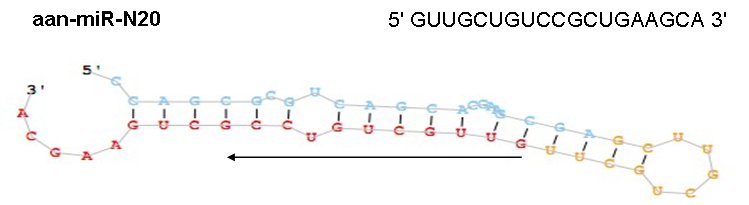


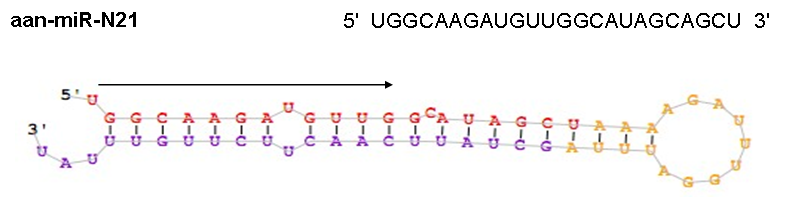


**Fig S1 The stem-loop structure of novel mosquito pre-miRNAs**

Arrows point to the mature miRNA sequences from 5' to 3'.
