# Supplementary material for: Identification and characterization of the expression profile of microRNAs in Anopheles anthropophagus
Source: Parasit Vectors. 2014 Apr 1;7:159. doi: 10.1186/1756-3305-7-159 (PMC4022070; doi:10.1186/1756-3305-7-159)
Supplement: Additional file 3: Table S2 — The GC content of the microRNA (miRNA) in Northern blot. [file 1756-3305-7-159-S3.doc]

| **Name** | **Sequence** | **GC%** |
| --- | --- | --- |
| hsa-miR-141 | UAACACUGUCUGGUAAAGAUGGC | 43.5 |
| hsa-miR-200c | UAAUACUGCCGGGUAAUGAUGGA | 43.5 |
| aan-miR-1000 | AUAUUGUCCUGUCACAGCAGU | 42.9 |
| aan-miR-184 | UGGACGGAGAACUGAUAAGGGC | 54.6 |
| aan-miR-989 | UGUGAUGUGACGUAGUGGUAC | 47.6 |
| aan-miR-2943 | UUAAGUAGGCACUUGCAGGCAAA | 43.5 |

Table S2 the GC content of the microRNA (miRNA) in Northern blot

Note: hsa-miR-141 and hsa-miR-200c are low GC miRNAs in Kim's article [34]
