# Supplementary material for: Identification and characterization of the expression profile of microRNAs in Anopheles anthropophagus
Source: Parasit Vectors. 2014 Apr 1;7:159. doi: 10.1186/1756-3305-7-159 (PMC4022070; doi:10.1186/1756-3305-7-159)
Supplement: Additional file 4: Figure S2 — Expression patterns of four mosquito-specific miRNAs isolated with mirVana miRNA isolation kit. [file 1756-3305-7-159-S4.doc]

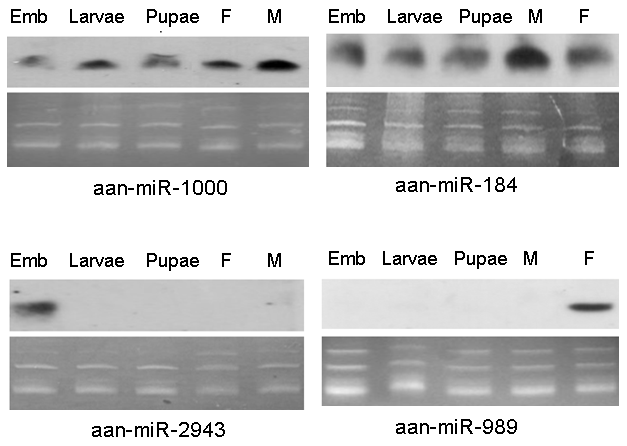


**Figure S2 Expression patterns of four mosquito-specific miRNAs isolated with** **mirVana miRNA isolation kit.**

The top panels are northern results and the bottom panels are RNA gel images for verification of small ribosomal RNA and tRNA integrity and loading of total RNA. Emb, pooled embryos between 0-36 hr after egg deposition; Larvae; mixed instar larvae; Pupae, mixed pupae; F, adult females one to five days after emergence; M, adult males one to five days after emergence. About 20 μg of total RNA were used per sample.
